# Does sex moderate health and Alzheimer's disease risk tied to early educational experiences? The reducing inequities through social and educational change follow-up in early adulthood extension study protocol

**DOI:** 10.1016/j.bbih.2026.101294

**Published:** 2026-06-27

**Authors:** Saché M. Coury, Savannah D. Lopez, Paul W. Savoca, Elizabeth M. Gaines, Brandon Parenti, Alondra Razon, Kulwant K. Dosanjh, Jennifer S. Labus, Jonathan P. Jacobs, Teal S. Eich, Mitchell D. Wong, Bridget L. Callaghan, Jennifer A. Silvers

**Affiliations:** a Department of Psychology, University of California, Los Angeles, Los Angeles, CA, USA; b David Geffen School of Medicine at UCLA, Los Angeles, CA, USA; c Goodman-Luskin Microbiome Center, University of California, Los Angeles, Los Angeles, CA, USA; d Vatche and Tamar Manoukian Division of Digestive Diseases, Department of Medicine, David Geffen School of Medicine at UCLA, Los Angeles, CA, USA; e Division of Gastroenterology, Hepatology and Parenteral Nutrition, Veterans Affairs Greater Los Angeles Healthcare System, Los Angeles, CA, USA; f G. Oppenheimer Center for Neurobiology of Stress and Resilience, University of California Los Angeles, Los Angeles, CA, USA; g Brain Research Institute, Gonda (Goldschmied) Neuroscience and Genetics Research Center, University of California Los Angeles, Los Angeles, CA, USA; h Leonard Davis School of Gerontology, University of Southern California, Los Angeles, CA, USA

## Abstract

Alzheimer's disease (AD) –a progressive neurodegenerative disorder that is characterized by insidious cognitive decline and distinct neuropathological features– significantly impacts daily life functioning and behavior and is disproportionally prevalent in women compared to men. The reasons and risk factors for sex-based disparities in AD prevalence are still largely unclear, however early life exposures (e.g., education and stress) may be important contributing factors. Therefore, it is increasingly important to disentangle the complex interactions between known early environmental protective and risk factors and genetic susceptibility and uncover how these factors might impact and shape neurobiological processes. Moreover, it is critical to assess how these processes, in turn, influence later cognitive and brain health outcomes that may confer sex-specific pathways of risk for developing AD. In this paper we describe the rationale and study protocol for The Reducing Inequities through Social and Educational Change Follow-Up in Early Adulthood Extension (RISE-Up EA+; R01AG089426) study, a follow-up study of 300 participants aged 24-26 years old that leverages a natural quasi-experimental cohort to investigate how health outcomes tied to socioeconomic mobility opportunity may contribute to sex-specific vulnerability for developing AD later in life. To examine how sex-specific vulnerabilities related to early educational experiences may set the stage for later AD risk, we will assess self-report, cognitive, biological (e.g., inflammation and microbiome), and brain health measures. Results from this work provide the opportunity to better understand how adolescent mobility opportunities might contribute to later life health outcomes and influence sex-specific developmental pathways important for later AD risk.

## Highlights

• Alzheimer's Disease (AD) significantly impacts cognitive, health, and social functioning.
• Women are disproportionately affected by AD compared to men.
• The underlying causes for sex-based disparities in AD are largely unknown.
• This study explores sex-specific developmental pathways important for AD risk.
• Results will inform how early mobility opportunity may shape later health outcomes.

## 1 Introduction

Alzheimer's disease (AD) is a progressive neurodegenerative disease characterized by significant cognitive decline that has serious impacts for health and social functioning in daily life. AD accounts for 60%-80% of the 50 million individuals living with dementia (Alzheimer's disease facts and figures, 2024)—a group of symptoms that include impaired cognitive skills in memory, language, and problem-solving. Furthermore, women are also disproportionately affected by AD compared to men (Fisher et al., 2018), making up two-thirds of AD cases (Alzheimer's disease facts and figures, 2024), even when taking lifespan differences into account (Alzheimer's disease facts and figures, 2024). Women also show steeper rates of episodic memory decline (Payami et al., 1994; Farrer et al., 1997), a hallmark clinical symptom of AD, and present with more severe AD-related brain pathology postmortem (Hu et al., 2021; Abu Raya et al., 2026). With the high prevalence of AD and related dementias expected to rise due to the aging population (Alzheimer's disease facts and figures, 2024), burdens from AD will also continue to increase for individuals, families and caregivers, health care and the economy. While extensive research has shed light on pathological mechanisms underlying AD, understanding the cause for sex-based disparities in AD prevalence is still a matter of debate, and the search for effective interventions remains elusive. Both human and animal studies suggest that early life exposures – including both education and stress – may play a pivotal role in shaping the trajectory of late-life health outcomes (Huang et al., 2023; Lesuis et al., 2018), and may do so differently in males and females (Nianogo et al., 2022a; Yan et al., 2018). Therefore, one particularly fruitful area for investigation may be the way that sex interacts with factors that decrease (e.g., education) or increase (e.g., stress, poverty, and obesity) risk for AD across the lifespan given that evidence suggests these factors do not operate independently (Crimmins, 2020). Understanding these intersections will be essential to explaining the persistent disparities in AD and identifying more effective, tailored intervention strategies since AD risk is not evenly distributed in the population.

### 1.1 Promise of education in increasing resilience and reducing risk pathways for AD

Identifying modifiable risk factors and developmental pathways that can inform prevention and early intervention are extremely important for improving AD outcomes. While there are many examples of environmental risk and resilience factors for AD (Zhang et al., 2021), one that is particularly pertinent to disease risk is education. High-quality and long duration of education are cornerstones of cognitive development, and thus serve as critical and modifiable resilience/risk factors for AD and dementia (Livingston et al., 2017; Seblova et al., 2023). In general, education plays an important role in building cognitive resilience and reserves (Livingston et al., 2017; Stern and Barulli, 2019; Stern et al., 2023; Stern, 2012), which help the brain to withstand pathological changes associated with AD and maintain function for a more extended period during the lifespan, potentially postponing symptom onset.

Moreover, education interacts with other facets of individual's lives, potentially moderating the impact of additional risk variables for AD, like lower socioeconomic status (SES) (Zahodne et al., 2024; Marden et al., 2017; Russ et al., 2013), which is typically indexed by income, education and occupation. Early poverty and lower educational attainment are associated with increased rates of AD, and lower cognitive functioning in later adulthood (Trani et al., 2022; Arce Rentería et al., 2019; Sisco et al., 2015; Eng et al., 2021; Kobayashi et al., 2019). Yet, upwardly mobile youth who “strive” to rise above poverty (i.e., youth striving to improve their socioeconomic status or climb the socioeconomic ladder, driven by factors such as greater educational attainment) typically have better cognitive outcomes in early adulthood (Turrell et al., 2002; Wong et al., 2022), which may ameliorate the impact of poverty on elevated AD risk (Turrell et al., 2002; Wong et al., 2022). We use the term “mobility opportunity” in this paper to describe the probabilistic, rather than deterministic, opportunity for upwards mobility in youth with higher quality educational experiences. That is, as a result of these better educational experiences, these youth have an enhanced potential for greater socioeconomic attainment, which may or may not be realized for each individual. We discuss this term in the context of early educational experiences—youths afforded the opportunity for high quality educational experiences by random lottery admission into high achieving charter schools compared to those who did not win the lottery (i.e., were waitlisted) and did not attend these schools. Mobility opportunity in the context of the present study protocol conveys that youth who won the random lottery were afforded the opportunity to strive for greater socioeconomic attainment by attending these higher performing schools. Thus, higher quality educational experiences, particularly in the context of low SES, may comprise a resilience pathway to reduce risk for later AD.

Some of the impacts of education on lowering AD risk may also be realized through changes to health behaviors and chronic disease risk. Specifically, greater educational attainment (e.g., high school graduation) is associated with slower pace of aging (even after accounting for genetic factors and tobacco smoking) (Sugden et al., 2023), better health behaviors and lower rates of chronic diseases (Cutler and Lleras-Muney, 2006, 2010; Galama et al., 2017; Lleras-Muney, 2022), and adolescents attending high-performing schools are less likely to engage in risky substance use or have substance using peers in their social network (Dudovitz et al., 2018), and report lower rates of hazardous or dependent alcohol use and lower cannabis misuse scores (Wong et al., 2022) compared to those in lower-performing schools. Not only are health behaviors directly linked to AD risk, worse health can also interfere with educational attainment and performance (Hysenbegasi et al., 2005; Taras and Potts-Datema, 2005), further impacting AD risk.

### 1.2 Education as a double-edged sword for health outcomes

While education access is generally associated with positive health outcomes, striving for upward mobility can also come with health costs for youth from low SES backgrounds (Chen et al., 2022). Prior observational studies indicate that youth from low SES backgrounds who have high academic achievement are at greater risk for a host of health challenges, including higher rates of diabetes (Brody et al., 2016), metabolic syndrome (Gaydosh et al., 2018), susceptibility to viral infection in adulthood (Miller et al., 2016), higher levels of inflammation (Loucks et al., 2010; Na-Ek and Demakakos, 2017; Castagné et al., 2016), and higher allostatic load (Chen et al., 2015; Brody et al., 2013). One reason for the negative health effects associated with academic striving in low SES individuals might be that youth from these backgrounds, who are also often racial and ethnic minorities, are more likely to experience social stressors within high-performance schools lacking diversity (i.e. both racial/ethnic and SES diversity) (Chen et al., 2022). These stressors could be a result of social isolation, uncertainty of social status, and internal identity conflicts, as well as potentially racism, discrimination, and feeling out of place (Johnson et al., 2011; Hardaway and Mcloyd, 2009; Destin, 2019). Additionally, some studies have shown evidence for links between upward mobility and increased perceived stress (Miller et al., 2020; Wong et al., 2026) and elevated rates of reported mental health problems in upwardly mobile youth compared to those with stable, high SES backgrounds (Islam and Jaffee, 2024) depicting this potential for mobility-related health tradeoffs. Prior evidence shows goal-striving related stress—defined as the discrepancy between goals and achievement while weighting the disappointment associated with not achieving one's goals—predicts increased health risks including higher risk for obesity (Cain-Shields et al., 2022a), kidney disease (Cain et al., 2019; Cain-Shields et al., 2021), mental health outcomes (Neighbors et al., 2011), and cardiovascular disease (Glover et al., 2020). Thus, the evidence to date suggests that youth from low SES backgrounds striving for upward mobility may face a double-edged sword with regards to health outcomes. Therefore, it is important to consider the costs of upward mobility on health outcomes as these costs may impede education as a protective factor against AD risk, especially among racial/ethnic diverse samples (Gaydosh et al., 2018; Miller et al., 2016; Brody et al., 2013; Chen et al., 2019).

### 1.3 Sex as a moderator of the impacts of education on risk for AD

Notably, while education and higher SES are well-established protective factors for AD, such associations may be complicated when considered in the context of sex. Importantly, two older studies both found that women with fewer years of education were at higher risk for AD compared to women with more years of formal education, an association that was absent in men (Letenneur et al., 2000; Launer et al., 1999). These results, though preliminary, suggest that the buffering effects of education on AD risk may be differentially expressed as a function of sex, and particularly important for women. However, there is a dearth of research testing this hypothesis formally (Liu et al., 2022), underscoring the need for more work in this space. At the same time, recent findings from a randomized education intervention study in majority low-SES youth show that while both females and males in the intervention group (i.e., lottery winners assigned to attend a high-performing school) were both more stressed regarding school performance and school/leisure balance compared to the control group (i.e., waitlisted youth), females in the intervention group experienced increased self-reported perceived stress (i.e., subjective current stress in life more broadly) while striving for higher education during adolescence and early adulthood (Wong et al., 2026). However, this was not true for striving males as less perceived stress was reported among males in the intervention group (Wong et al., 2026), indicating gender differences in striving-related stress responses (i.e., stress associated with higher performance education experiences). These results align with findings from the Jackson Heart Study in African American adults which show observed gender differences in stress outcomes with women reporting higher goal-striving stress compared to men in the sample (Glover et al., 2020; Cain-Shields et al., 2022b). Notably, additional observed associations from the Jackson Heart Study between goal-striving related stress and increased health risks appear to be only significant for women and not men (Cain-Shields et al., 2022a; Glover et al., 2020). Few studies have explicitly reported on sex or gender differences in striving-related psychosocial and health outcomes, but when such differences have been observed, they have generally indicated worse outcomes for females compared to males (Wong et al., 2022, 2026; De France et al., 2022). One possibility for such findings is that females striving for upward mobility may be especially vulnerable to stress due to gendered social expectations. For instance, societal expectations or norms that position women as primary caregivers can conflict with expectations in school environment/institutional demands in educational settings, creating stress. This explanation is preliminarily supported by results that show females striving for higher education (i.e., won the random lottery to attend high-performing schools) also reported significant stress at home, stress of peer pressure, and stress of financial pressure compared to females that were waitlisted to attend high-performing schools (Wong et al., 2026). This suggests that the protective effects of education against later life health outcomes and AD risk might be moderated by sex, particularly for individuals from low SES backgrounds. Additionally, given that stress has been associated with and is a risk factor for the development and progression of AD (Zhu et al., 2021; Donley et al., 2018; Lyons et al., 2022; Song et al., 2020), reducing the physiological consequences from striving-related stress may improve cognitive health trajectories later in life. Therefore, it is crucial to assess interactive factors, such as sex, and biological mediators that inform resilience and risk pathways. In particular, it is vital to understand how such stress can impact sex-associated health outcomes, such as AD, and identify factors, particularly early in life before cognitive decline or core brain pathology, including β-amyloid (Aβ) and tau pathology, are detectable, to inform prevention strategies and mitigate risk.

### 1.4 Sex interactions with health risk factors linked to AD

Obesity is one of the top modifiable risk factors for AD in the United States (Nianogo et al., 2022b) and more prevalent among women than men (Koceva et al., 2024), making it especially important to consider in terms of sex-related risk. Importantly, obesity significantly increases the risk of AD as much as 6-fold additive risk in predictive models (Kivipelto et al., 2005). However, the greater risk for AD linked to obesity is especially heightened in women – e.g., in older women, every unit increase in body mass index (BMI) is associated with a 36% increase in AD risk (Gustafson et al., 2003). Obesity not only interacts with sex, but also other risk and resilience factors for AD including education and upwards mobility. Specifically, in a prior randomized education intervention study in majority low-SES youth (Wong et al., 2014, 2022), it was shown that females randomized to attend a high-performing public charter high school had higher rates of overweight and obesity than females randomized to attend the lower-performing school (19.3% difference in rates between groups), as well as worse self-reported physical health (Wong et al., 2022; Dudovitz et al., 2018). The opposite trends were reported in males – randomization to the higher-performing schools were associated with lower rates of overweight and obesity (−14.28% difference in rates) and better self-reported physical health (9.67% difference), relative to males randomized into the lower performing schools (Wong et al., 2022; Dudovitz et al., 2018). The randomized nature of this study allows us to conclude that high quality education, previously considered a universal protective factor against AD, may in fact increase the incidence of the top modifiable risk factor for AD – obesity – in low SES females. This unintuitive and alarming finding further highlights the essential need to study how sex interacts with other important risk and protective factors for AD across the lifespan.

### 1.5 The brain-gut axis as a conduit for health behavior impacts on AD risk

While the evidence for some health outcomes and behaviors acting as risk factors for AD is clear, the biological pathways via which those risks operate is not. One especially important set of biological pathways to consider in the context of striving-related stress, health outcomes and sex are inflammation and the microbiome, both operating and modulated through the wider brain-gut axis. Critically, both inflammation and the microbiome are linked to cognition (Spyridaki et al., 2016; Leigh and Morris, 2020; Koblinsky et al., 2023), and specifically to AD. (Askarova et al., 2020; Vogt et al., 2017; Bhattacharjee and Lukiw, 2013; Kinney et al., 2018; Akiyama et al., 2000; Lee et al., 2010) The microbiota and inflammation also exhibit strong reciprocal connections, which appear to operate through changes in intestinal permeability and microbial metabolites, amongst other mechanisms (Hantsoo et al., 2019; Honda and Littman, 2012; Strober, 2013; Boer et al., 2019; Clemente et al., 2018). Moreover, experimental models have shown that microbial impacts on cognition operate through changes to inflammatory processes (Shen et al., 2020; Kheirvari et al., 2022).

Chronic low grade inflammation, particularly neuroinflammation through increased inflammatory mediators, such as pro-inflammatory cytokine expression in the brain, is a hallmark symptom of AD that exacerbates AD pathogenesis and progression (Duarte-Guterman et al., 2020; Kloske and Wilcock, 2020). Previous studies have demonstrated a clear role for inflammation in AD, including microglial activation and increased neuroinflammation (ElAli and Rivest, 2016; Cai et al., 2022; Hashioka et al., 2021) as well as elevated peripheral pro-inflammatory markers invoking systemic inflammation ultimately affecting brain health and different characteristics of AD pathogenesis in preclinical and clinical cases (Xie et al., 2021; Zhang et al., 2025; Cai et al., 2025; Holmes et al., 2009; Leung et al., 2013; Motta et al., 2007; Anuradha et al., 2022). In addition, preliminary research in mouse models for AD have identified sex-specific differences in microglial function, potentially modulated by hormonal changes/variations and genetic risk (Saha and Sisodia, 2024), that may contribute to varied manifestations of AD between females and males. Specifically, rodent models have revealed that female microglia transition more rapidly to a pro-inflammatory, disease-associated state compared to males, which is marked by higher expression of mRNAs associated pro-inflammatory gene expression in microglia (Saha and Sisodia, 2024; Stephen et al., 2019; Guillot-Sestier et al., 2021). This pro-inflammatory microglial profile (disease-associated microglia) has been correlated with reduced compaction and deposition of Aβ plaques as well as decreased ability and effectiveness to clear away and remove Aβ plaques (Saha and Sisodia, 2024; Stephen et al., 2019; Guillot-Sestier et al., 2021). Female mice have also exhibited earlier microglial activation, Aβ deposition, and behavioral changes compared to male mice (Saha and Sisodia, 2024; Gallagher et al., 2013; Lynch, 2022). One recent study using translocator protein position-emission-tomography imaging with human AD patients found that females had a stronger Aβ-plaque-independent microglial response, which was significantly associated with tau pathology, but did not show any differences in Aβ-plaque-dependent microglial response compared to males (Biechele et al., 2024). These findings provide valuable preliminary insight into sex-specific differences for neuroinflammation in AD pathogenesis and it will be important to continue to determine potential translational differences in human populations as well.

Obesity is a risk factor for AD and is associated with chronic low-grade systemic inflammation, cognitive impairment, and AD-related brain pathology (i.e., reduced white matter) in late middle-aged adults (Alford et al., 2018). Increased peripheral pro-inflammatory response from adipose tissue in obese individuals can lead to chronic systemic inflammation, which can increase the permeability of the blood-brain-barrier, allowing pro-inflammatory cytokines to cross from the periphery to the brain, thereby impacting brain health and cognition (Spyridaki et al., 2016; Alford et al., 2018). Obesity is also linked to increased pro-inflammatory gene expression (Brunelli et al., 2021; Lee et al., 2020; Coín-Aragüez et al., 2018) and higher circulating levels of pro-inflammatory cytokines, such as interleukin-6 (IL-6) and Tumor Necrosis Factor alpha (TNF-α) (Eder et al., 2009; Calder et al., 2011; Tzanavari et al., 2010). Additionally, sex appears to moderate the link between obesity and neuroinflammation in AD, such that higher BMI in women with AD was shown to be significantly associated with a stronger Aβ-plaque-independent microglial response, but this was not true for males with AD. (Biechele et al., 2024) Further elucidating the role of inflammation and its contributions to AD, particularly earlier in life, would help to inform the development of preventative strategies and therapeutic interventions that can target such modifiable risk factors in a sex-specific manner.

While less is known about how the microbiome links to AD, relative to inflammation, the microbial links appear to manifest both in gut microbiome community composition (Vogt et al., 2017; Zhuang et al., 2018) and microbial functional pathways (Liu et al., 2019) that are distinct in AD patients compared to healthy-aged controls. Gut microbiota produce bacterial amyloids that can cross-seed the misfolding of proteins in the brain (Askarova et al., 2020; Kowalski and Mulak, 2019), contributing to and enhancing Aβ plaque aggregation. These bacterial products can also induce microglial priming increasing neuroinflammation that promotes a further cascade of AD pathogenesis (Askarova et al., 2020; Kowalski and Mulak, 2019). Additionally, obesity has been linked to significant changes in the diversity and composition of the microbiota in the gut (Dong et al., 2020, 2022; Gupta et al., 2020; Singer-Englar et al., 2019), which could push it towards a higher AD-pathology promoting state. Moreover, sex-specific hormones may play an important role in modulating gut microbiota influencing sex differences in AD pathogenesis. Animal models have previously shown that changes in female hormone levels can influence microbiome composition and microbiota function, as well as microbiota can in turn regulate hormonal levels that may influence important diseases mechanisms, such as Aβ levels (Saha and Sisodia, 2024). Given these findings, studies identifying the microbiome links with AD, particularly in sex-specific ways, are essential future research steps especially given the higher prevalence of AD among women.

### 1.6 Sex differences influence genetic risk for AD

Beyond modifiable risk factors for AD, sex appears to interact with a known genetic risk factor (Ungar et al., 2014; Holland et al., 2013; Altmann et al., 2014; Damoiseaux et al., 2012) for AD that can be assessed well before aging occurs: the *ε4* form of the Apolipoprotein E (*ApoE*) gene (Alzheimer's disease facts and figures, 2024). Relative to *ApoE*-ε4 carrying males, *ApoE*-ε4 carrying females have higher risk of earlier AD onset (Payami et al., 1994; Farrer et al., 1997; Aggarwal and Mielke, 2023), worse cognitive outcomes and performance (Aggarwal and Mielke, 2023; Raber et al., 1998; Arenaza-Urquijo et al., 2024; Leung et al., 2012; Andrews-Zwilling et al., 2010), even earlier in life (Reynolds et al., 2019), and appear to have brain health (Aggarwal and Mielke, 2023; Sampedro et al., 2015) and pathology (Cacciottolo et al., 2016; Hohman et al., 2018; Shokouhi et al., 2020) impacted differently. Interestingly, both inflammation and the microbiome are associated with *ApoE* alleles. Specifically, the *ε4* high risk variant has been associated with altered microbiome composition and increased neuroinflammation in both mice and humans (Kloske and Wilcock, 2020; Tai et al., 2017; Tzioras et al., 2019; Tran et al., 2019; Zajac et al., 2022). Several recent studies in both mice and humans have also demonstrated that sex and *ApoE-ε4* interact to affect the microbiome and inflammation (Duarte-Guterman et al., 2020; Seo et al., 2023; Maldonado Weng et al., 2019; Ayton et al., 2021). Together, these data suggest that a mechanistic understanding of sex differences in AD risk will emerge only when examining *ApoE-ε4*, the microbiome, and inflammation together in the context of educational opportunities.

### 1.7 Towards a comprehensive understanding of sex-related AD risk

The research reviewed so far highlights that female sex might be related to higher AD incidence by virtue of its interactions with several known risk and resilience factors for AD – genetic variation, education and upwards mobility – and that some of these impacts may be realized, especially earlier in life, via functioning across the brain-gut axis. As such, an understanding of sex-related risk for AD will only emerge with a comprehensive analysis that includes all of these variables assessed in development, before AD pathology and cognitive decline is evident. Given that the majority of these prior findings are based on observational study designs, causal designs where protective and risk factors are experimentally manipulated are invaluable. The Reducing Inequities through Social and Educational Change Follow-Up in Early Adulthood Extension study (RISE-Up EA+; R01AG089426) will address these issues by providing a unique developmental and experimental framework for understanding the impacts of sex on AD risk, especially via pathways that interact with striving-related stress and may set the stage for brain-gut axis changes which foreshadow later emerging cognitive and brain-pathology.

**Fig. 1:**
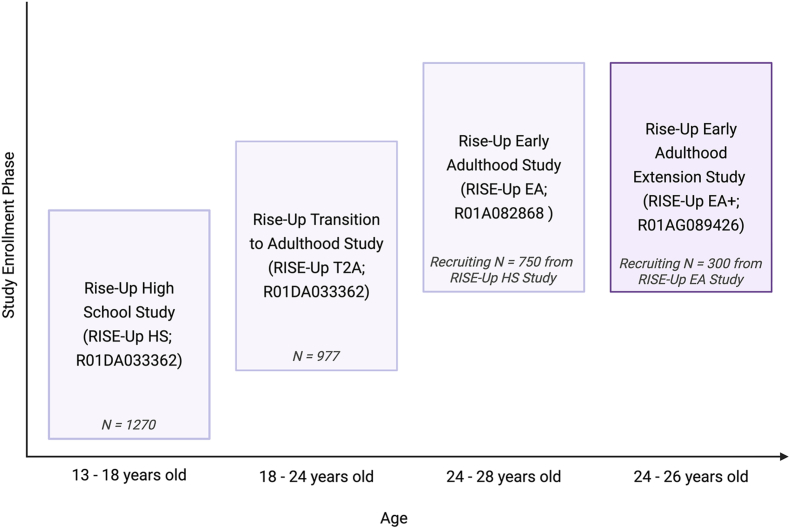
*Timeline of RISE-Up Study Phases*. 1270 participants were recruited and enrolled at baseline in the RISE-Up High School Study and were followed throughout grades 9-12. 977 participants continued with the RISE-Up Transition to Adulthood Study and were followed through age 24. The RISE-Up into Early Adulthood Study phase is ongoing with the goal of recruiting 750 participants ages 24-28 that previously participated in the baseline study. The RISE-Up Early Adulthood Extension Study is ongoing with the goal of recruiting 300 participants ages 24-26 from the RISE-Up EA Study. Created in https://BioRender.com.

The RISE-Up EA+ study leverages a natural quasi-experimental cohort that began in 2013 (RISE-Up) to understand the impact of educational experience on youth health outcomes and substance use (R01DA033362). The random admissions lottery of five high-performing charter schools in Los Angeles was initially used to find two comparable groups of adolescents that were exposed to high-and lower-performing public high schools. These groups had similar baseline demographics, prior academic performance, family characteristics, and neighborhood exposures, and have been followed longitudinally during adolescence from ages 13 through 18 (phase 1: RISE-Up-HS; R01DA033362), during their transition to emerging adulthood from ages 18 through 24 (phase 2: RISE-Up-T2A; R01DA033362), and into early adulthood from ages 24 through 28 (RISE-Up EA; R01A082868) while collecting various self-reported health metrics to understand the impact of education on health outcomes (see Fig. 1 for a timeline of study phases). (Wong et al., 2014, 2022) To assess cognitive ability and brain health, as well as associated inflammatory and microbiome-related biology, we will conduct an add on to the RISE-Up EA study (called RISE-Up EA+) to collect additional cognitive and neurobiological measures after participants began enrollment in RISE-Up EA when the cohort is aged between 24 and 26 years. Findings from earlier timepoints in the RISE-Up study position it to address the unique question of how sex-related AD risk may manifest across the lifespan. Specifically, earlier waves from grade 9 through grade 11 (RISE-Up-HS) identified that adolescents attending high-performing schools were less likely than those in lower-performing schools to engage in risky substance use and have substance using peers in their social network (Dudovitz et al., 2018). Recent results also show that young adults aged 20 and 21 (RISE-Up-T2A) that attended high-performing schools had lower rates of hazardous or dependent alcohol use, had lower cannabis misuse scores, less delinquent behaviors, and higher rates of fair/poor mental health scores (Wong et al., 2022). Differences in physical health outcomes were found between males and females, where rates of fair/poor physical health and overweight/obesity were lower for young adult males that attended high-performing schools but higher rates were found for young adult females (Wong et al., 2022). Controlling for academic outcomes (e.g., high school graduation and grade point average, standardized test scores and college matriculation) had no impact. This may indicate that the developmental pathways for later AD risk may already be in motion for females with greater mobility opportunity in the RISE-Up study (Wong et al., 2022). However, the precise biological mechanisms informing this risk are still unclear. In this paper, we will summarize the study aims, hypotheses, and protocol for the RISE-Up EA+ Study that aims to understand how factors contributing to health after mobility opportunity impact sex-related risk for developing AD later in life.

## 2 Study aims & hypotheses

**Fig. 2:**
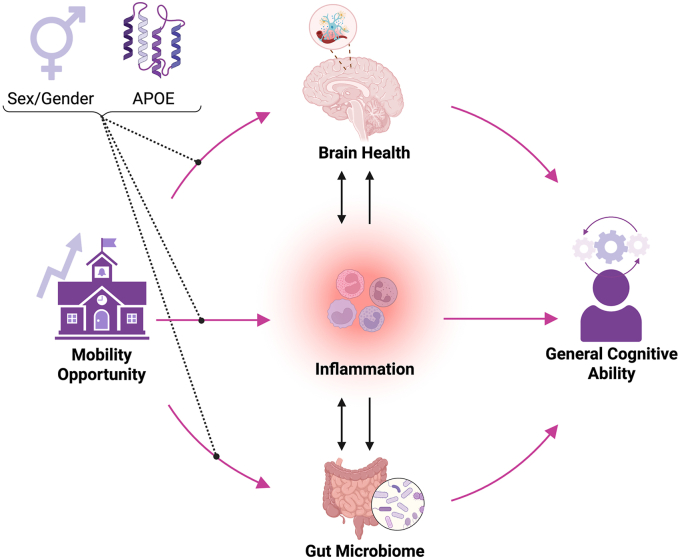
*The RISE-Up EA+ Conceptual Model.* The conceptual model outlines study analysis plans to 1) examine if sex moderates the association between mobility opportunity, brain health and general cognitive ability; 2) investigate links between mobility opportunity and health mechanisms (inflammation and microbiome), and whether those links are moderated by sex; 2a) conduct an exploratory analysis to test whether sex and *ApoE*-ε4 interact to predict health profiles (proinflammatory blood and stool profiles); 3) assess if brain health and health mechanisms (inflammation and microbiome) mediate the link between mobility opportunity and general cognitive ability and whether this is moderated by sex. Created in https://BioRender.com.

In the RISE-Up EA+ Study, we will collect measures of striving-associated stress through self-report. We will quantify health risks by indexing brain-gut axis function via inflammatory markers in blood, the microbiome metagenome in stool, *ApoE* gene allele via buccal swab, brain health through Magnetic Resonance Imaging (MRI), and general cognitive ability through standardized neurocognitive tests. Using these measures, we will examine how sex moderates the links between youths afforded mobility opportunity by randomization into high performance schools vs. those who were not (i.e., waitlisted) and brain health, general cognitive ability, inflammatory markers, and the microbiome (see Fig. 2 for the conceptual model). Both sex assigned at birth and gender identity might contribute to potential differences observed in this study and we will aim to try to parse these constructs when we can, highlighting that sex-related differences may be, in part, due to stress related to gender roles and expectations. The aims of our study are as follows:

1. **Identify the impact of mobility opportunity on young adult general cognitive ability (GCA) and brain health (which we propose will link with later life resilience to AD), and determine if those associations are moderated by sex.** We hypothesize (H1) that mobility opportunity will be associated with worse brain health in females, but better brain health in males, relative to static opportunity. Second, we hypothesize (H1.2) that mobility opportunity will be associated with greater GCA in both males and females, relative to static opportunity.
2. **Establish links between mobility opportunity, inflammatory markers, and microbiome enrichment for inflammatory genes and pathways, and determine whether those links are moderated by sex and sex-related vulnerabilities (adolescent stress).** We expect (H2) that relative to static opportunity, mobility opportunity in females (but not males) will have a greater average expression of inflammatory genes in blood, and genes and pathways involved in inflammation in stool.

In addition to our primary Aim 2, we also have an exploratory Aim 2 to test whether sex and *ApoE*-ε4 status interact to predict inflammatory markers and microbiome enrichment for inflammatory genes and pathways, especially in females. For the exploratory analysis, we hypothesize (H3) that *ApoE*-ε4 carrying mobility opportunity females will have the most proinflammatory blood and stool profiles.

3. **Determine whether the association between mobility opportunity and GCA is mediated by brain health, inflammatory blood markers, and the microbiome (parallel mediators) and moderated by sex.** We hypothesize (H4) that females exposed to mobility opportunity during adolescence, relative to males exposed to mobility opportunity, will have more pro-inflammatory profiles in blood and stool (increased physical health risks), and worse brain health (diminished activation in prefrontal areas supporting cognitive function) during early adulthood, which will in turn mediate the association with GCA in early adulthood. We expect to see these sex differences indicating sex-specific-pathways within the mobility opportunity group, reflecting greater impact of wear and tear over time from mobility opportunity in females compared to males. In turn, we propose that these may be early markers for increased AD risk later in life that may be realized over time. However, within females at this age, we still expect to see that mobility opportunity females will have better GCA relative to static opportunity females.

## 3 Methods

### 3.1 Recruitment & eligibility

**Fig. 3:**
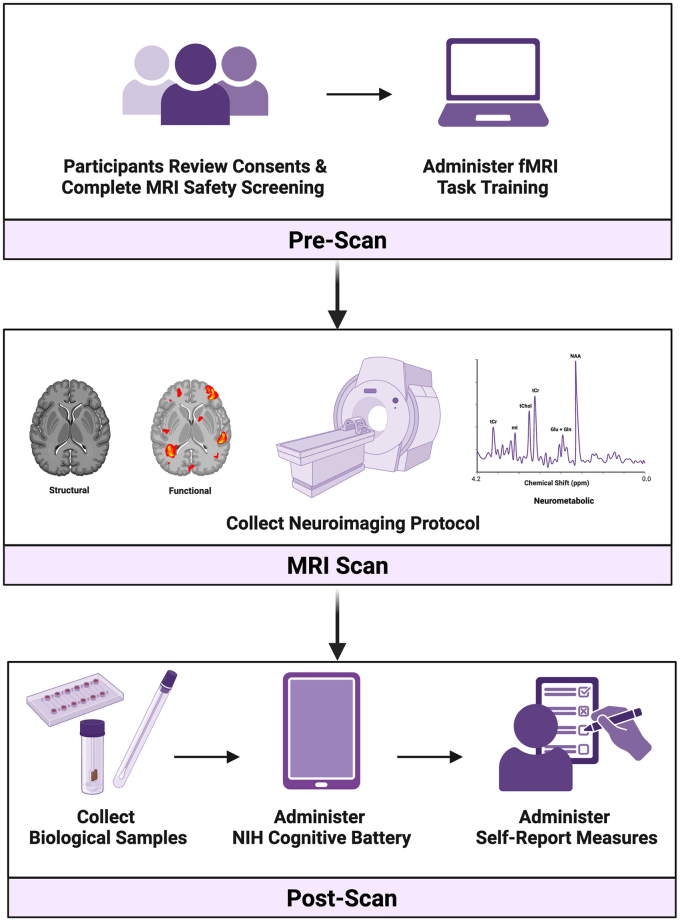
*The RISE-Up EA+ In-Person Session Flow.* Eligible participants will be scheduled for in-person sessions at UCLA. During the pre-scan section of the session, participants will review consent forms, complete updated MRI safety screening forms, and will be trained on fMRI tasks that they will complete inside the scanner. During the MRI scan, we will collect multimodal imaging data including structural, functional, and neurometabolic data. After the scan, participants will contribute biological samples, complete the NIH-Toolbox, and provide self-report questionnaires if these were not completed prior to the in-person session. Created in https://BioRender.com.

The demographics of the original RISE-Up Study have been described extensively elsewhere (Wong et al., 2022; Dudovitz et al., 2018), but broadly consisted of 1270 participants (52.6% female, 47.4% male, median age 14.2) who were entering 9th grade in Fall 2013 or Fall 2014. Participants for the original study were largely recruited from and are representative of low-income neighborhoods in Los Angeles, with 1137 (89.5%) identifying as Latino (Dudovitz et al., 2018), and all applied for high-achieving charter high school admission lottery, with 694 students (54.6%) being admitted on the basis of random lottery (intervention/mobile group) and 576 students (45.4%) on the waiting list (control/static group). Recruitment for the RISE-Up-EA+ extension study is dependent upon previous participation in the RISE-Up-EA follow up study, which is ongoing (see Fig. 1 for a timeline of study phases). A subsample of 300 participants aged 24-26 years old who previously consented to follow-up contact will be included in the extension study. Detailed contact information has been maintained for prior participants, enabling digital communication via text message and email. Interested respondents will schedule a phone screening, during which a study coordinator will administer a brief screening interview and obtain informed consent. During this phone screen, the research staff will also explain procedures for the extension study. Participants who meet eligibility criteria for the study (including MRI safety screening) and agree to participate will be scheduled for a three and a half hour on-site in-person session at the University of California, Los Angeles (UCLA) where they will complete a magnetic resonance imaging (MRI) scan, behavioral tasks, cognitive assessments, and will be administered biospecimen samples (see Fig. 3 for session flow). Prior to their in-person session, participants will be consented and will be administered questionnaires to complete remotely or they will have the opportunity to complete questionnaires at the in-person session. The protocol for this study has been approved by the Medical Institutional Review Board of the University of California, Los Angeles (IRB#24-000281).

*Exclusion Criteria:* Exclusionary criteria include typical MRI contraindications (e.g., the presence of braces or metal implants). Due to the requirements of an in-person study visit, previously-enrolled participants who have relocated out of state will be excluded. Since the original RISE-Up study's inception, only 30 participants (2.4%) have moved entirely out of California.

### 3.2 Measures

#### 3.2.1 Self-report questionnaires

A range of assessments have been collected and examined in the original RISE-Up cohort since baseline across multiple longitudinal timepoints in the following domains that will be used to supplement the extension study, including assessments examining socioemotional factors, important life events and SES factors, health behaviors and conditions, arrest and incarceration data, and risk taking. In addition to these measurements, the RISE-Up-EA+ extension study will also administer a range of self-report surveys to participants assessing stressful exposures, mental health, and physical health below.

##### 3.2.1.1 Stressful exposures

Early life adversity will be assessed using the Childhood Trauma Questionnaire (CTQ) which assesses physical, emotional and sexual abuse and physical and emotional neglect in 70 items scored on a 5-point Likert scale (α = .95) (Bernstein et al., 1994, 2003). Current stress will be assessed using the 10-item Perceived Stress Scale (PSS), which asks participants to rate how unpredictable, uncontrollable, and overloaded they found their lives over the last month on a 5-point Likert scale (α > .84) (Cohen et al., 1983). Additionally, we will assess discrimination experiences using the 9-item Everyday Discrimination Scale, which asks participants to rate the frequency of discriminatory actions experienced, and what characteristic they attribute those actions to (α = .63 for lifetime and .80 for everyday). (Krieger et al., 2005; Williams et al., 2003; Taylor et al., 2004).

##### 3.2.1.2 Emotional and mental health

Mental health will be assessed using a short form of the Mood and Anxiety Symptom Questionnaire (Mini-MASQ - 26 items), which asks respondents to rate the extent to which they experienced negative affect, positive affect, and somatic arousal, across the last week on a 5-point Likert scale (Casillas and Clark, 2020). Emotion regulation strategy usage will be assessed using the 22-item Extended Emotion Regulation Questionnaire (E-ERQ) which measures respondents’ tendency towards cognitive reappraisal, expressive suppression, distraction, selective attention, and situation selection, on a 7-point Likert scale (Guassi Moreira et al., 2024). Trait strategy usage scores will be computed by separately averaging items for each of the five strategies.

##### 3.2.1.3 Physical health

Health-related quality of life will be assessed using the RAND 36-item Short Form Health Survey Instrument (SF-36), which measures domains of physical function, role disability due to physical and emotional problems, bodily pain, general health perceptions, vitality, social function, and general mental health (α > .75) (Jenkinson et al., 1994; McHorney et al., 1993; Brazier et al., 1992). To assess diet, we will use Vioscreen, a gold-standard, web-based, image-assisted, Food Frequency Questionnaire (Kristal et al., 2014), which compiles 90 days of dietary data, including what a participant has ate and drank over this time period. Dietary outputs include a personal health and nutrition summary, personal health and nutrition report, a health eating index food feedback report, top foods report, and a dietary inflammatory index report and relevant data will be used as covariates in analyses involving the microbiome data. At the in-person session, we will also collect data on participant height, weight, and BMI. Prior to the in-person session, we will obtain information on medication use, antibiotic use, supplements, alcohol use and smoking history. These variables will likely be used as covariates in analyses examining the microbiome and inflammation data.

#### 3.2.2 General cognitive ability

The NIH Toolbox behavioral cognitive assessment (Weintraub et al., 2014; Mungas et al., 2014) is a series of seven cognitive batteries that will be administered on an iPad during the on-site study visit. This assessment will be used to determine individual cognitive construct assessment scores across each of the seven tasks as well as used to determine summary scores for total cognition composites. The total cognition composite scores (Heaton et al., 2014) that get computed will serve as a measure of adult general cognitive ability (GCA) in our analyses. The NIH Toolbox includes multiple cognitive tasks that will be administered and used to comprise the total cognition composite score from measures of cognitive flexibility (*Dimensional Change Card Sort*), attention (*Dimensional Change Card Sort* & *Flanker Inhibitory Control and Attention*), inhibitory control (*Flanker Inhibitory Control and Attention),* episodic memory (*Picture Sequence Memory Tests),* pattern processing speed (*Pattern Comparison Processing Speed Test),* receptive vocabulary (*Picture Vocabulary Test)*, working memory (*List Sorting Working Memory Test)*, as well as crystallized abilities and reading decoding skills (*Oral Reading Recognition).*

#### 3.2.3 Neuroimaging

Participants will complete their on-site in-person session at the UCLA Staglin Center for Cognitive Neuroscience (CCN). All neuroimaging scans (structural, functional, and neurometabolic sequences) will be performed on a Siemens MAGNETOM 3T scanner (Siemens Healthcare GmbH, Erlangen, Germany) with a Nova Medical 32-channel head coil. Please see below for a summary of each type of scan sequence acquisition (see Table 1 for a summary list of all scan parameters; all sequences are described in greater detail below).

**Table 1:** Summary overview of scan acquisitions and basic parameters.

Scan Acquisition	Voxel Size (mm^3^)	Averages	Slice #	FOV (mm)	TR/TE/TI (ms)	Flip Angle (°)	Scan Time	Scan Type
***Structural MRI***								
**T1-weighted MPRAGE**	1.0x1.0x1.0	1	208	256	1900/2.48/900	9.0	4:33	Grant
**Neuromelanin**	0.4x0.4x2.5	2	11	220	750/12/NA	120	3:44	Exploratory
***Functional MRI***								
**N-back fMRI task**	3.4x3.4x4.0	1	33	220	2000/30/NA	75	3:00/run	Grant
**Rule Switching fMRI task**	3.4x3.4x4.0	1	33	220	2000/30/NA	75	6:04	Grant
**Reward Learning fMRI task**	3.4x3.4x4.0	1	33	220	2000/30/NA	75	6:06/run	Exploratory
**Emotional Movie fMRI task**	3.4x3.4x4.0	1	33	220	2000/30/NA	75	8:00	Exploratory
**Resting-State fMRI**	3.4x3.4x4.0	1	33	220	2000/30/NA	75	7:00	Exploratory
***Neurometabolic Imaging***								
**dACC sLASER SVS MRS**	15x36x12	64	1	NA	2000/30/NA	90 excitation/180 refocusing	2:26	Grant
**L & R Hippocampus sLASER SVS MRS**	15x20x10	128	1	NA	2000/30/NA	90 excitation/180 refocusing	4:34	Grant

##### 3.2.3.1 Structural magnetic resonance imaging

###### 3.2.3.1.1 T1-weighted anatomical MRI

A T1-weighted magnetization-prepared rapid gradient-echo (MPRAGE) sequence will be acquired. This acquisition will be used to prescribe voxel placement to improve estimations of structural accuracy for the magnetic resonance spectroscopy (MRS) sequences, will be used during pre-processing and registration of the functional MRI (fMRI) scans, and will undergo structural processing to obtain metrics for different brain tissues. Gray matter (GM) volumes and white matter (WM) hyperintensity, which are early indicators of cognitive decline (Tondelli et al., 2012; Debette and Markus, 2010; Yamasaki et al., 2021; Raz et al., 2012), will be assessed using FreeSurfer (Knussmann et al., 2022; Fischl et al., 2002) and ENIGMA (Grasby et al., 2020) protocols to implement structural processing, extract metrics, and perform quality control procedures.

##### 3.2.3.2 Task-based functional magnetic resonance imaging

###### 3.2.3.2.1 Working memory & cognitive control task

The Letter N-back fMRI task will be used to assess working memory and cognitive control. This validated task demonstrates age-related differences in performance accuracy and activation of frontoparietal control networks in younger and older adults (Yaple et al., 2019), as well as between young adults who differ in terms of *ApoE* genotype (Scheller et al., 2017). This task has also been shown to differentiate between adults (without AD) who varied in terms of their reports of memory issues, suggesting that this task could be a helpful early predictor of AD. (Yeung et al., 2022) Participants will complete a blocked version of the N-back task with a cognitive load (2-back) and a control condition (0-back) (Jaeggi et al., 2010; Wang et al., 2019; Redick and Lindsey, 2013). For each block, participants will complete 20 trials, each involving seeing a letter presented for 1500 ms with a jittered interstimulus interval. On 0-back (control) trials, participants are instructed to press a key with their index finger whenever they view the letter “X.” On 2-back trials (high working memory load), participants are instructed to press with their index finger whenever they see a letter that matches the letter they viewed two trials ago. Participants are instructed to press with their middle finger for all “non-match” trials. There are 60 trials per condition across three runs of this game, with each run lasting 3 min.

###### 3.2.3.2.2 Cognitive flexibility task

We will assess cognitive flexibility by administering the Rule Switching fMRI task, which differentially recruits frontostriatal networks across age in young adulthood (Rubia et al., 2006). This task leverages a rapid, mixed trial, random presentation, event-related design and is 6 min and 4 s long (152 total trials with jittered ITI). Participants will view a 2x2 grid with a double headed arrow positioned either horizontally or vertically at the center of the grid. On each trial, a red dot will appear in one of the squares of the grid and participants are instructed to respond about whether the red dot is on the right or left of the grid if the arrow is positioned horizontally, and whether the red dot appears in the top or bottom of the grid if the arrow is positioned vertically. Switch trials (when the rules change) are randomly dispersed after four, five, or six repeat trials (when the rule stays the same).

##### 3.2.3.3 Neurometabolic imaging

###### 3.2.3.3.1 Magnetic resonance spectroscopy (MRS)

Neurometabolic data will be characterized with single voxel (SV) magnetic resonance spectroscopy (MRS) in three different regions: dorsal anterior cingulate cortex (dACC), right hippocampus, and left hippocampus. The dACC was chosen as a region of interest because of its involvement in cognitive control and it has been previously implicated in MRS studies assessing metabolic alterations in those with mild cognitive impairment and AD. (Huang et al., 2017; Chen et al., 2025) The hippocampus was chosen as a region of interest given that it is a critical region for learning and memory and it has been shown to be a sensitive and central region for AD, particularly for disease pathology, early disease diagnosis, as well as for disease progression (Song et al., 2021). Additionally, prior MRS studies have observed significant metabolite alterations in the hippocampus in AD patients compared to controls, including metabolites that are indicators of neuroinflammation (Song et al., 2021). In line with MRS expert consensus (Wilson et al., 2019) for data acquisition at 3T *in vivo*, we will implement a short echo time semi-LASER (sLASER) sequence (Oz and Tkáč, 2011; Deelchand et al., 2021) to be used with an automated B_0_ and B_1_ shimming calibration protocol (Gruetter and Tkác, 2000; Deelchand et al., 2022) to improve acquisition of high-quality MRS data. LCModel (Provencher, 2009) will be used to fit and quantify all estimated metabolite concentrations with total creatine acting as an internal reference standard. Quality assurance procedures include ensuring all metabolite estimations that will be used for analysis have relative Cramer-Rao lower bounds (CRLBs) < 25%. GM and WM tissue percentages will also be calculated within each voxel to correct for total tissue content.

#### 3.2.4 Additional measures for exploratory analyses on emotion and reward

Emotion processing dysfunction (i.e., facial and social emotion processing) and disturbance (Chaudhary et al., 2022; Spoletini et al., 2008; Weiss et al., 2008; Jiskoot et al., 2021; Stam et al., 2023), have been previously associated with AD, with emotion recognition deficits manifesting at earlier stages in the course of AD. (Chaudhary et al., 2022) Preliminary evidence has also shown evidence for reward processing deficits in AD. (Perry and Kramer, 2015) Importantly, anxiety and depression can manifest at various stages of AD (Botto et al., 2022), but are often early behavioral symptoms and signs of AD related dementia development (Alzheimer's disease facts and figures, 2024; Botto et al., 2022). Thus, assessing emotion and reward related processing may be particularly important for early detection of AD related dementia. As such, this study protocol will include multiple exploratory indices of neural and behavioral measures of emotion and reward processing (described in this section). Half of the participants in this cohort (N = 150) will be randomly assigned to complete the additional emotion processing tasks (emotional movie fMRI task and faces behavioral task) and the other half will be randomly assigned to complete the additional reward related tasks (reward learning fMRI task and post-scan reward memory task). We will also aim to collect neuromelanin and resting state functional imaging on as many participants as possible as part of the scan protocol if time allows. Please see below for additional exploratory imaging protocols.

##### 3.2.4.1 Additional task-based functional magnetic resonance imaging

###### 3.2.4.1.1 Reward learning task

150 participants will be randomly assigned to complete the reward learning fMRI task (Esfand et al., 2024), adapted from existing reward learning paradigms that successfully engage frontostriatal regions (Calabro et al., 2023; Parr et al., 2021), inside the scanner to assess reward-related brain activation and connectivity. This task has two phases: the first is the reward learning phase which is completed during the fMRI task inside of the scanner and the second is the reward spatial memory phase which is completed as a post-scan computer task. Participants will complete two 6 min and 6 s runs (36 trials per run) with the same sequence parameters as the task-based functional imaging in Table 1.

In brief, during the reward learning phase, participants will explore a 3x3 grid map by choosing different locations on the map to move a penguin to find potential hidden rewards. For each trial, participants have two options of where they can move their penguin; these options are first depicted as “#”s (2-6s) that are then replaced by a “1” or “2”. The participant will have a brief amount of time (2s) to select either location 1 with their index finger or location 2 with their middle finger to move and display the penguin in the new location on the map (2s). If they do not select a location, they will be automatically moved to location 1. Next, the trial outcome will be displayed (2s) for the participant to receive feedback: no reward (blank), small reward (one gold coin; 75% of rewards), or a large reward (multiple stacked gold coins; 25% of rewards). Each map location will have a set reward probability (25%, 50%, or 75%) while each option presented to move to will always differ in their likelihood of reward (see Esfand et al., 2024 for task design figures).

After the scan, participants will complete the reward spatial memory task to assess how much they learned. This computer task consists of 27 trials with no performance feedback. Participants will be shown the same 3x3 grid map but will now indicate which of the two map location options (indicated with a “1” or “2” on the computer) were associated with a greater probability of rewards (i.e., their goal will be to choose the location with the larger reward outcome) (Esfand et al., 2024). Following their location selection, a “∗” will be shown to confirm their selection choice (2s).

###### 3.2.4.1.2 Emotional movie task

150 participants will be randomly assigned to complete the emotional movie task that will present participants with a brief suspenseful clip from the “Bang! You're Dead” episode of the “Alfred Hitchcock Presents” television series (1961). The adapted movie clip shown to participants in the scanner is an 8 min condensed compilation of the original 25 min episode that still preserves the episode's essential plot narrative and has been successfully used in previous research studies (Shafto et al., 2014; Geerligs and Campbell, 2018; Keles et al., 2024; Taylor et al., 2017; Kliemann et al., 2022; Sun et al., 2022). During the scan, participants will be instructed to watch, listen and continuously (sampled at ∼ 10 Hz) report their subjective experience of emotional intensity while viewing the video. The rating of intensity will not have bounds: the reported ratings can increase or decrease without limit. Participants will indicate increases (more) or decreases (less) in intensity using two buttons that allow the subjective intensity rating line at the bottom of the screen to change throughout the movie. Participants will not be aware of the movie title prior to viewing it and will be asked after viewing the movie if they have seen this movie previously. This 8 min scan will have the same sequence parameters as the task-based functional imaging presented in Table 1.

##### 3.2.4.2 Additional structural magnetic resonance imaging

###### 3.2.4.2.1 Neuromelanin

We will collect a T1-weighted Fast Spin Echo sequence to estimate levels of neuromelanin, a byproduct of norepinephrine metabolism (Clewett et al., 2016) and a proxy measure of dopamine (Wakamatsu et al., 2015; Horga et al., 2021), in dopaminergic and noradrenergic rich brain regions including the substantia nigra and the locus coeruleus—important regions that show neurochemical changes in response to emotional stress (Tanaka et al., 2000), are important for cognitive processing (Clewett et al., 2016; Sara, 2009; Caestecker et al., 2025), and are affected by AD brain pathology early (Caestecker et al., 2025; Beardmore et al., 2021) which may contribute to early anxiety and depression symptoms (Iannitelli et al., 2023). This sequence will utilize the following parameters: TR = 750ms; TE = 12.0ms; FA = 120°; FOV = 220mm; Slice thickness = 2.5mm; Voxel size = 0.4x0.4x2.5mm^3^; Scan time = 3 min 44 s (see Table 1). The FOV will be manually aligned to be centered on the locus coeruleus prior to collecting.

##### 3.2.4.3 Resting-state functional magnetic resonance imaging

###### 3.2.4.3.1 Resting-state

We will collect resting-state fMRI to assess whole brain functional organization and connectivity with the same imaging parameters as the task-based functional imaging in Table 1. Participants will be instructed to remain awake and stare at a fixation cross on the screen during the 7 min sequence.

##### 3.2.4.4 Behavioral tasks

###### 3.2.4.4.1 Faces task

We will collect a computerized behavioral faces task that consists of angry, happy and surprised face stimuli drawn from the racially diverse affective expression (RADIATE) dataset (Conley et al., 2018). This task design is adapted from prior work investigating valence bias and behavioral markers of tolerance of ambiguous threat (VanTieghem et al., 2017; Saragosa-Harris et al., 2023). The behavioral task design for this study follows the behavioral categorization task design specifications from Saragosa-Harris et al. (2023) with minor language changes to the valence labels. In brief, participants will view surprised (ambiguous), angry (negative), and happy (positive) faces across ten blocks (10 surprised, 5 happy, and 5 angry faces in each block; stimulus presentation for 500ms) and will be asked to indicate whether the face/person in the image is “positive” or “negative” by pressing a button on the keyboard (“1” or “0”, counterbalanced; response screen prompt 1500ms). After each trial response, there will be a 200ms fixation cross and in between each block there will be a 10 s fixation cross. Participants will be trained with feedback prior to completing this task using six practice trials (2 happy, 2 angry, and 2 surprised) that include novel images from a separate dataset (Tottenham et al., 2009). Feedback will not be included in the actual task.

#### 3.2.5 Biospecimen samples & measurements

##### 3.2.5.1 Inflammation

Non-fasting dried blood spot samples will be collected in the RISE-Up-EA follow up study using Tasso M-20 and T-20 microfluid devices (Tasso, Inc., Seattle, WA, USA) (Wixted et al., 2022). The RISE-Up-EA follow up study will also collect self-report medical history, acquire current medication usage, and health behaviors such as alcohol, smoking, and drug use. If blood samples were not collected at the follow up study (RISE-Up-EA), participants will have the option to provide blood samples at this extension study (RISE-Up-EA+) during the in-person visit. If blood is collected at the extension study visit, we additionally collect information on recent illness and/or vaccines during sample collection. Once collected, samples will be dried, transferred to a biohazard bag with a desiccant, and stored in a −80° Celsius freezer until they are ready to be assayed to preserve protein and nucleic acid biomarkers. These samples will be assayed for pro-inflammatory gene expression profiles, specifically conserved transcriptional response to adversity (CTRA), as well as C-reactive protein (CRP), a systemic peripheral marker of inflammation. Importantly, we are not assessing acute inflammatory responses, effects, or reactivity. Instead, we are identifying more “trait-like” immune profiles by leveraging gene expression and circulating markers indicating systemic inflammation to understand striving-related stress experiences and health-related mechanisms.

##### 3.2.5.2 Microbiome

Gut microbiome samples will also be collected in this study. We will administer the OMNIgene OMR-200 stool collection kits (DNAGenotek) to index gastrointestinal microbiome. These stool samples will be collected at home and mailed back to the lab. Briefly, participants will collect a pea size amount of stool using a toilet hat and spatula. A bead homogenizes the sample into a solution which stabilizes the DNA at ambient temperature for 60 days. Once mailed back to the lab, the sample will be stored in a −80° Celsius freezer until samples are ready for DNA extraction and sequencing.

##### 3.2.5.3 ApoE genotype

Buccal cell samples will be collected using a buccal swab kit (BHD400b) from Boston Heart Diagnostics in order to assess *ApoE* genotype. The dried blood spot samples are not used to assess *ApoE* genotype because all of the wells are needed for immunoassays for CTRA and CRP, which is why we collect a buccal cell sample. Immediately after collection, samples will be mailed directly to Boston Hear Pre-Analytics. Participants will be classified dichotomously based on the number of *ε4* alleles: none (*ε4-*) versus 1 or 2 (*ε4+*). *ε2/ε4* participants will be excluded from analysis due to the opposing associations between the *ε2* and *ε4* alleles and cognition (Berge et al., 2014).

##### 3.2.5.4 Supplemental health measure: oral microbiome

While not part of the initial study design, we will collect an oral microbiome sample as a supplemental health measure to assess an additional pathway where educational experiences might modify health outcomes given preliminary evidence showing that communities of oral microbes and bacteria (Liu et al., 2023; Guo et al., 2023) as well as periodontal inflammatory metrics (Melo et al., 2025) have been altered in AD. OMNIgene OM-501 samples (DNAGenotek) will be administered during the in person visit after the scan ensuring the participants have not ingested food or water for at least 30 minutes prior to the sample. Samples will be incubated at 50 °C for 2h, vortexed, aliquoted into cryogenic tubes and then frozen at −80 °C after these preprocessing steps in lab to prepare them for shipment to the sequencing site. In addition, we will administer an adapted version of the Oral Health Questionnaire (Simpson et al., 2020) to participants to assess overall oral health, including oral hygiene behaviors and symptoms, in order to compute a total oral hygiene score (Buunk-Werkhoven et al., 2011) to be included as a covariate in analyses.

## 4 Power analysis

We conducted a power analysis where tests of hypothesized differences were performed using linear contrast analysis within the framework of random effects general linear models (GLMs). For these single degree of freedom tests, power was approximated using two-tailed independent sample *t*-test in G∗Power 3.1.9.7. For the moderator analysis examining sex-specific associations between education opportunity and the primary outcome, brain health, at an alpha .05/2 markers of brain health = .025, 150 participants per group (mobile/static 1:1 ratio female:male) provided adequate power (80%) to detect an effect size difference as small as Cohen's d = 0.36 (tcrit = 2.25, noncentrality parameter = 3.10), if it exists. This sample size also provided 80% power to detect associations as little as f^2^ = .04 (multivariate linear regression for moderated mediation analyses, Crit F = 2.24, noncentrality parameter = 13.07).

## 5 Discussion

This study provides the opportunity to utilize a quasi-experimental framework to begin to understand how sex-specific vulnerabilities may be associated with educational opportunities in adolescence that will impact physical health risks and cognition in early adulthood, ultimately setting the stage for AD risk later in life. We expect that sex-specific vulnerabilities related to mobility opportunity in adolescence will increase physical health risks in early adulthood: increased inflammation and altered microbiome, specifically in females. We hypothesize that, in turn, a female's physical health risks will be related to their cognitive performance in young adulthood and to the neural underpinnings of those cognitive skills, revealing early indicators of AD risk after education opportunities. In other words, we suggest that the differential impact of high-performance schooling on physical health in females and males may undo the cognitive resilience generated through better education in females. Additionally, we will explore how education opportunities in adolescence interact with genetic vulnerabilities for AD - *ApoE-ε4* – for example, it is possible that striving-related stress may undo some of the cognitive benefits of educational opportunity via health pathways in females, which may in turn relate to their cognitive outcomes and neural correlates therein. By investigating the impact of schooling on inflammation and microbiome profiles in young adulthood, this study will reveal potential developmental mechanistic pathways that might be linked and contribute to AD outcomes much later in life.

Striving for better educational attainment during adolescence can be stressful for youth, seemingly more so for females, and this striving-related stress may induce consequential health problems, such as increased systemic inflammation, altered brain-gut axis signaling and other neurotoxic effects, including neuroinflammation, impacting later cognitive outcomes and AD risk. An overarching goal of this study is to critically consider and examine the potential health costs from psychosocial factors—such as stress and educational attainment—in diverse youth early in life. By pursuing this research, we may be able to assess in the future whether intervening and improving factors earlier in development, such as education and educational settings by improving and increasing support and resources while youth are striving, can ultimately impact and set the stage for health outcomes across the lifespan and later illness risk. It is critically important to note that our findings would reveal what kinds of support are needed, particularly for females early in life, to ensure such pursuits for improved future socioeconomic attainment do not evoke unintended and deleterious health consequences. In addition to increasing and maintaining cognitive reserves by improving education attainment as an important and feasible early prevention strategy for AD, prior work has also identified that reducing risk for neuropathological damage is important for slowing progression and prevention (Zhang et al., 2021). Therefore identifying early indicators and biological mechanisms of AD and related dementias is important for modulating disease development and outcomes. The presence of A β plaques and neurofibrillary tangles are both core features of the brain pathology that characterizes AD. Understanding the biological mediators, such as peripheral and central inflammation and altered gut-brain-axis signaling, that impact onset, generation and progression of these pathologies in AD is important to improve health outcomes and reduce risk.

A strength of the RISE-Up study is its quasi-experimental design through random admissions lottery to several charter schools allowing us to sample comparable cohorts of adolescents exposed to different school environments and thus, more rigorously test the causal effect of exposure to high-performing schools on health and cognition. This design avoids the limitations of observational studies assessing health and education and allows for more control over confounding variables, such poverty, neighborhood, and family factors (Cutler and Lleras-Muney, 2006, 2010; Galama et al., 2017). One limitation of the data collected thus far in the RISE-Up cohort is its reliance on self-reported health data. The addition of objective markers of health (e.g., microbiome and inflammation) in the follow up timepoints will greatly add to the rigor of this study and expand the research scope. Furthermore, little is known about risk factors for AD that may be unfolding during young adulthood despite the knowledge that this is a pivotal period for physical and mental health. In general, more research is also needed to determine more consistent evidence for sex and gender differences in risk pathways for AD. However, the research conducted thus far highlights the urgent need to understand how and why potential modifiable risk factors modulate the risk and progression of AD, and how they can be leveraged to reduce AD risk for all. Therefore, it is critically important to assess how sex interacts with potential mechanisms that contribute to increased health risks, especially earlier in life, that may inform vulnerabilities for AD. Therefore, this study has unprecedented opportunity to identify mechanisms and explicitly assess sex-specific vulnerabilities that may in turn be used to identify optimal windows for prevention or invention efforts that optimize cognitive and physical health outcomes in youth striving for upward mobility from low socioeconomic status backgrounds.

The RISE-Up EA + study adds an additional timepoint of data collection to the longitudinal RISE-Up Study in early adulthood with neurobiological data targeting the brain-gut axis, to allow us to rigorously test causal effects of exposure to high-performing schools on health, brain-gut signaling, and cognition outcomes in early adulthood, which may set the stage for later AD risk. The overall goals of this project are to elucidate how adolescent mobility opportunities will be tied to later cognitive and brain health outcomes in young adulthood via sex-specific changes to potential biological mediators—inflammation and gut-brain-axis—to inform vulnerabilities for developing AD later in life. This study seeks to discern causal links between education and health by taking an innovative approach by assessing early developmental pathways that may set the stage and be linked to AD risk later in life. By identifying early markers before AD manifests, we can work to improve resilience pathways and decrease risk pathways to improve health outcomes and functioning in later adulthood. Supporting youth socioeconomic development and socioeducational success is extremely important. This research aims to find modifiable factors to improve health outcomes and support youth early in life so that everyone can reap the benefits that high quality education has to offer while reducing potential negative health consequences that may accompany sex-specific development.

## Funding

This research is supported by the NIH-NIA National Institute on Aging (1R01AG089426-01 to JAS and BLC), the UCLA Cousins Center for Psychoneuroimmunology (PNI) - Psychoneuroimmunology Seed Grant (to BLC and MDW), and the Iris Cantor-UCLA Women's Health Center/CTSI Pilot Award (UL1TR001881 to JAS).

## CRediT authorship contribution statement

**Saché M. Coury:** Investigation, Methodology, Project administration, Software, Visualization, Writing – original draft, Writing – review & editing. **Savannah D. Lopez:** Investigation, Project administration, Writing – review & editing. **Paul W. Savoca:** Investigation, Project administration, Software, Writing – review & editing. **Elizabeth M. Gaines:** Project administration, Software, Writing – review & editing. **Brandon Parenti:** Investigation, Writing – review & editing. **Alondra Razon:** Investigation, Writing – review & editing. **Kulwant K. Dosanjh:** Writing – review & editing. **Jennifer S. Labus:** Funding acquisition, Writing – review & editing. **Jonathan P. Jacobs:** Funding acquisition, Writing – review & editing. **Teal S. Eich:** Funding acquisition, Writing – review & editing. **Mitchell D. Wong:** Funding acquisition, Writing – review & editing. **Bridget L. Callaghan:** Conceptualization, Funding acquisition, Methodology, Supervision, Writing – original draft, Writing – review & editing. **Jennifer A. Silvers:** Conceptualization, Funding acquisition, Methodology, Supervision, Writing – original draft, Writing – review & editing.

## Declaration of competing interest

The authors declare that they have no known competing financial interests or personal relationships that could have appeared to influence the work reported in this paper.

## Data Availability

No data was used for the research described in the article.
